# Chromosomal microarray testing in adults with intellectual disability presenting with comorbid psychiatric disorders

**DOI:** 10.1038/ejhg.2016.107

**Published:** 2016-09-21

**Authors:** Kate Wolfe, André Strydom, Deborah Morrogh, Jennifer Carter, Peter Cutajar, Mo Eyeoyibo, Angela Hassiotis, Jane McCarthy, Raja Mukherjee, Dimitrios Paschos, Nagarajan Perumal, Stephen Read, Rohit Shankar, Saif Sharif, Suchithra Thirulokachandran, Johan H Thygesen, Christine Patch, Caroline Ogilvie, Frances Flinter, Andrew McQuillin, Nick Bass

**Affiliations:** 1 Division of Psychiatry, University College London, London, UK; 2 North East Thames Regional Genetics Service Laboratory, London, UK; 3 Nottinghamshire Healthcare NHS Foundation Trust, Nottingham, UK; 4 Kent and Medway NHS and Social Care Partnership Trust, Kent, UK; 5 East London NHS Foundation Trust, London, UK; 6 Surrey and Borders Partnership NHS Foundation Trust, Surrey, UK; 7 Camden and Islington NHS Foundation Trust, London, UK; 8 Cumbria Partnership NHS Foundation Trust, Cumbria, UK; 9 South West Yorkshire Partnership NHS Foundation Trust, Yorkshire, UK; 10 Cornwall Partnership NHS Foundation Trust, Cornwall, UK; 11 Southern Health NHS Foundation Trust, Southampton, UK; 12 Coventry and Warwickshire Partnership NHS trust, Coventry, UK; 13 Guy’s and St Thomas’ NHS Foundation Trust, London, UK

## Abstract

Chromosomal copy-number variations (CNVs) are a class of genetic variants highly implicated in the aetiology of neurodevelopmental disorders, including intellectual disabilities (ID), schizophrenia and autism spectrum disorders (ASD). Yet the majority of adults with idiopathic ID presenting to psychiatric services have not been tested for CNVs. We undertook genome-wide chromosomal microarray analysis (CMA) of 202 adults with idiopathic ID recruited from community and in-patient ID psychiatry services across England. CNV pathogenicity was assessed using standard clinical diagnostic methods and participants underwent comprehensive medical and psychiatric phenotyping. We found an 11% yield of likely pathogenic CNVs (22/202). CNVs at recurrent loci, including the 15q11-q13 and 16p11.2-p13.11 regions were most frequently observed. We observed an increased frequency of 16p11.2 duplications compared with those reported in single-disorder cohorts. CNVs were also identified in genes known to effect neurodevelopment, namely NRXN1 and GRIN2B. Furthermore deletions at 2q13, 12q21.2-21.31 and 19q13.32, and duplications at 4p16.3, 13q32.3-33.3 and Xq24-25 were observed. Routine CMA in ID psychiatry could uncover ~11% new genetic diagnoses with potential implications for patient management. We advocate greater consideration of CMA in the assessment of adults with idiopathic ID presenting to psychiatry services.

## Introduction

Intellectual disability (ID) is defined as significant impairments in intellectual and adaptive functioning with onset before the age of 18 years. ID is a clinically heterogeneous disorder with a range of genetic and environmental causes. Genetic causes include aneuploidies, copy-number variations (CNVs) and single-nucleotide variants in specific genes. There are a wide range of environmental causes, notably perinatal infection and hypoxic injury. For ~50% of individuals with ID the cause is unknown.^1^ There is an increased prevalence of psychiatric disorders in adults with ID, for example the point prevalence of psychosis has been estimated as 10 times higher than the general population.^2^ In the United Kingdom adult ID psychiatry is a specialist field. Investigation of the cause of ID/developmental delay (DD) predominately occurs at onset in childhood and there is no formalised system of diagnostic review. Genetic testing in adulthood can be carried out by ID psychiatrists and other treating clinicians, such as neurologists. Clinical genetics services are organised regionally in the UK and treating clinicians can make onward referrals for patients and families. Screening for genetic causes of ID has advanced from G-banded karyotyping to high-resolution genome-wide chromosomal microarray analysis (CMA), which is often the recommended first-tier cytogenetic test for DD/ID.^3^

CMA encompasses array comparative genomic hybridisation (aCGH) and single-nucleotide polymorphism arrays, which detect submicroscopic losses or gains of genetic material known as CNVs. The clinical relevance of CNV events is determined by factors such as the inheritance pattern, size of the CNV, number of genes deleted or duplicated, likely functional consequence of gene disruption and presence of the CNV in healthy control data sets.^3^ Recurrent CNVs are found in regions of the genome harbouring simple repetitive DNA sequences known as low copy repeats. Propensity to recombination errors in these regions give rise to some of the well-known syndromic forms of ID, for example the recurrent deletions at the 15q11-q13 region which cause Prader Willi or Angelman’s syndrome. CNVs can also be classed as pathogenic when there is a disruption of genes involved in important neurodevelopmental functions, such as neurotransmission.^4^

A CNV ‘morbidity map’ has identified 70 CNV regions significantly associated with DD/ID in 29 085 children; however, comparable data on adults with ID are limited.^4^ Study of research cohorts with schizophrenia and ASD have also revealed rare recurrent CNVs that are very strong risk factors for the development of these disorders.^5, 6^ It has now been shown that some CNVs confer risk for multiple neurodevelopmental disorders. For example duplication at the 16p11.2 locus increases risk for ID and ASD and has been shown to be associated with a 14-fold increased risk of psychosis.^7^ Thus rare recurrent CNVs have broad, and often comorbid, neurodevelopmental phenotypes operating across traditional diagnostic boundaries.

Historically genetic investigation has not been a routine part of the assessment of adults with ID presenting to psychiatric services.^8^ However, the emergence of CNVs as important risk factors for multiple disorders raises the possibility that individuals with comorbid neurodevelopmental conditions should be prioritised where CMA testing is offered. We investigated the presence of undiagnosed likely pathogenic CNVs in adults with idiopathic ID presenting to community and in-patient psychiatric services in England. We discuss the frequency and type of CNVs identified; the associated phenotype and the implications of our findings.

## Methods

### Study design and participant recruitment

Recruitment was undertaken via the Mental Health Research Network (MHRN) at 32 National Health Service (NHS) trusts and 1 non-NHS provider across England between August 2012 and March 2014. Consultant Psychiatrists in Intellectual Disabilities acted as local investigators at each site. Local investigators identified eligible participants from their caseloads based upon the study inclusion criteria, namely that the participants should be aged 18 years or older with idiopathic ID, one or more psychiatric diagnoses and/or significant challenging behaviours. Typically in the UK services use a high threshold for eligibility of ID services (IQ<70 as well as significant impairment of functioning which has been present from childhood). Idiopathic ID was defined as no clear genetic or environmental cause of ID detailed on the participant’s medical records. Capacity to consent to the research project was assessed in accordance with the Mental Capacity Act 2005. Easy read information sheets and consent forms were utilised with individuals who had capacity to consent. In the absence of capacity consultees were identified to give advice as to the person’s likely wishes regarding participation.

Clinical data including medical and psychiatric history (International Statistical Classification of Diseases and Related Health Problems (ICD-10) diagnoses), was collected from an informant and/or medical records. General observations for dysmorphic features were made and measurements of height and head circumference were collected by the clinician or researcher. Photographs were taken (where consent was given) for corroboration by the study team. Detailed psychiatric and behavioural phenotyping was undertaken using the Mini Psychiatric Assessment Schedule for Adults with Developmental Disabilities (Mini PAS-ADD) and Behaviour Problems Inventory - Short Form (BPI-S). The Mini PAS-ADD assesses psychiatric symptoms in seven diagnostic areas and provides threshold scores for symptoms that are likely to warrant a diagnosis in conjunction with a clinical assessment.^9^ The BPI-S provides frequency scores of behaviours on three domains, self-injurious behaviour, aggressive/destructive behaviour and stereotyped behaviour.^10^ Symptoms identified from Mini PAS-ADD or BPI-S screening are referred to as subclinical symptoms in the manuscript.

### Genetic analysis and feedback

Participants provided either a blood (24%) or saliva sample (76%) for DNA extraction. aCGH analysis was undertaken at the North East Thames Regional Genetics Service Laboratory on the Nimblegen 135K platform. Arrays were processed and CNVs were reported using clinical diagnostic laboratory protocols, in keeping with the Association for Clinical Genetic Science (ACGS) best practice guidelines.^11^ CNVs referred to in the manuscript as likely pathogenic include pathogenic causative findings and pathogenic susceptibility loci, both of which are thought to affect gene function in view of the associated phenotype.^12^ All likely pathogenic CNVs detailed in this manuscript were validated by qPCR, FISH or QF PCR and have been added to the Decipher genome browser.^13^ The other classes of CNV reported were variants of uncertain clinical significance (VOUS) and likely benign CNVs. Classification as VOUS includes: intronic CNVs, CNVs where no genes are present, CNVs where no entry was present on the Database of Genomic Variants (at the time of analysis), in addition to those where insufficient information is available to classify further. Further analysis has not been undertaken and these CNVs may include technical artefacts. Chromosomal abnormalities were also reported. Further analysis of relevant literature, and patient and control data sets was conducted by the research team and collaborators.

Likely pathogenic CNVs were fed back in writing to the participants’ treating psychiatrist. The cytogenetic report detailing relevant CNVs and associated publications were provided alongside chromosomal disorder guides from the support group Unique where available.^14^ There is a paucity of appropriate and accessible information for adults with ID receiving diagnoses. The study team developed easy to read materials to aid feedback for some of the clinically relevant CNVs. Psychiatrists also had the opportunity to speak with a member of the research team regarding the result prior to feeding this back to their patients and family members and/or carers. Referral to the regional clinical genetics service was recommended for all likely pathogenic results. There were no adverse outcomes reported from feedback of the genetic test results.

### Statistical analysis

Statistical analyses were undertaken using IBM SPSS Statistics for Windows, Version 22.0 (IBM Corp, Armonk, NY, USA). Univariate binary logistic regression was performed using Mini PAS-ADD thresholds and history of involuntary in-patient admission, including forensic in-patient section, as predictor variables. The binary outcome variable was presence or absence of a likely pathogenic CNV.

## Results

**Figure 1:**
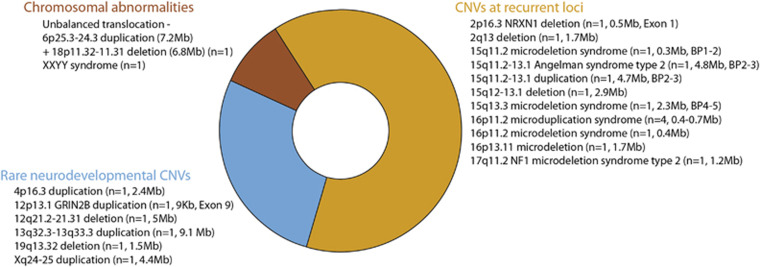
Overview of likely pathogenic CNVs identified in a sample of 202 adults with idiopathic intellectual disabilities and comorbid psychiatric disorders. Number of participants, approximate CNV size in megabases or kilobases (Mb or Kb), breakpoints (BP) and exon number are displayed in parenthesis as appropriate.

A total of 202 adults with idiopathic ID and comorbid psychiatric disorders/challenging behaviour were recruited to the study (63% male; mean age 37 years, range 18–78 years; 74% White British). The yield of likely pathogenic CNVs, including chromosomal abnormalities, was 11% (22/202). A further 62% of participants had a least one CNV classed as a VOUS (126/202) and 27% (54/202) had likely benign CNVs only. Details of the VOUS CNVs can be found in the Supplementary Information. An overview of likely pathogenic CNVs is presented in Figure 1, with detailed genetic and phenotypic data presented in Table 1 and Supplementary Table 1. A comparison of psychiatric diagnoses, subclinical symptoms and section history for likely pathogenic *versus* likely benign (including VOUS) CNVs is provided in Table 2. There were 21 participants on a forensic in-patient section at the time of recruitment and no other participants had a forensic section history. In all, 6/21 forensic in-patients carried likely pathogenic CNVs compared with 16/181 in participants not on a forensic in-patient section. Thus the proportion of likely pathogenic CNV carriers in forensic in-patients was higher with an OR of 4.1 (95% CI 1.40–12.04, *P*=0.01).

### Neurodevelopmental CNVs

Most of the likely pathogenic CNVs were observed in regions of the genome prone to recurrent CNV. Five of these CNVs were identified at the 16p11.2 locus (4 duplications, 1 deletion) and one duplication at 16p13.11. The 16p11.2 region is associated with increased risk for ASD, schizophrenia and major depressive disorder, in keeping with the phenotypes observed in this cohort.^15^ A further five CNVs were identified in the 15q11.2-13.3 region (15q11.2 deletion, Angelman syndrome type 2, 15q12-13.1 deletion, 15q11.2-13.1 duplication and 15q13.3 deletion) with variable psychiatric phenotypes. The CNVs at 15q11.2, 15q13.3, 16p11.2 and 16p13.11 affect neurosusceptibility loci. These likely pathogenic CNVs have incomplete penetrance in that they occur at higher frequencies in disease cohorts, however, are not always associated with a disease phenotype and can sometimes be observed in healthy controls.

Another region prone to recurrent CNV is the 17q11.2 locus, which encompasses the *neurofibromatosis type 1* (*NF1*) tumour suppressor gene. We identified a participant with a *NF1* microdeletion presenting with a clinical diagnosis of ASD and challenging behaviours. This supports previous evidence of ASD being associated with variants in the *NF1* gene.^16^ We also identified a deletion at 2q13 in a patient with a clinical diagnosis of ASD. Of the 29 patients described with this CNV 4 are reported to have ASD.^17^ CNVs in this region have also been shown to be enriched in schizophrenia cohorts,^18^ the participant presented with subclinical features of psychosis in addition to anxiety and behavioural problems. Two CNVs were identified in genes known to be important in neurodevelopment, namely GRIN2B and NRXN1. The *GRIN2B* gene is located at 12p13.1 and encodes the NR2 subunit of a *N*-methyl-*D*-aspartate glutamate receptor heteromer, which mediates excitatory neurotransmission and is thought to have an important role in memory and learning. Variants in the *GRIN2B* gene have previously been associated with behavioural problems.^19^ We identified a duplication affecting exon 9 of the *GRIN2B* gene in a participant displaying self-injurious and aggressive behaviours. The *NRXN1* gene is located at 2p16.3, it encodes a cell-surface receptor which is important for neurotransmission. Exonic NRXN1 deletions have been associated with increased risk for schizophrenia and ASD.^20^ Our participant has a deletion of exon 1 of the *NRXN1* gene, a clinical diagnosis of personality disorder and subclinical symptoms of psychosis and stereotyped behaviour.

Five of the likely pathogenic CNVs identified have little or no prior association with psychiatric phenotypes in existing literature and likely represent rare emerging neurodevelopmental CNVs. First, we identified a duplication of 4p16.3, a region where deletions give rise to the better characterised Wolf–Hirschhorn syndrome. The CNV identified in our sample partially overlaps with the CNV reported in a case study of a patient with attention-deficit hyperactivity disorder (ADHD).^21^ Our participant had a clinical diagnosis of ASD and was a forensic in-patient. A duplication at Xq24-25 was observed in a participant with aggressive and stereotyped behaviours. Abnormal behaviours, primarily hyperactivity, have previously been associated with CNVs in this region. The *STAG2* gene, which encodes a component of the cohesion complex and is essential for chromosome segregation in dividing cells, has been identified as the likely causative gene.^22^

We identified a participant with a deletion at 12q21.2-21.31 comprising 17 genes. This region contains the *Synaptotagmin-1* (*SYT1*) gene, which encodes a calcium-binding synaptic vesicle membrane protein involved in triggering neurotransmitter release at the synapse. A variant in *SYT1*, with a dominant negative function, has recently been associated with profound cognitive impairment.^23^ Whilst we observe a copy-number loss and different phenotype, a low haploinsufficiency score is suggestive of adverse functional consequences.^24^ The participant has a clinical diagnosis of paranoid schizophrenia, a history of alcohol abuse and was a forensic in-patient. Another participant has a duplication at 13q32.3-13q33.3 comprising 33 genes. This region contains the *D-amino acid oxidase activator* (*DAOA*) gene, which indirectly affects glutamatergic transmission and dopamine turnover. We and others have reported *DAOA* to be associated with both schizophrenia and bipolar affective disorder.^25^ The participant has a clinical diagnosis of ASD and ADHD, and is a forensic in-patient. Finally, we identified a deletion at 19q13.32 comprising 56 genes. This deletion partially overlaps with a case reported previously but does not include any of the proposed candidate genes.^26^ The participant has a clinical diagnosis of depression and bipolar affective disorder.

## Discussion

Approximately 11% new diagnoses could be made by testing adults with ID accessing psychiatry services in the UK. Recently there have been calls for increased clinical use of CMA in patients with schizophrenia. The yield of likely pathogenic CNVs in adults with schizophrenia is reported to be in the range of 2.5–5%.^27, 28^ Our higher diagnostic yield argues that clinicians should also consider adopting routine CMA in ID psychiatry. Interestingly the most frequently observed CNV in this study was the 16p11.2 duplication (4 individuals, 2%). This CNV has been widely reported in other studies, with a frequency of ~0.2% in DD/ID,^29^ ~1% in ASD^5^ and ~0.3% in schizophrenia.^28^ Accepting the small sample size this may suggest a particular enrichment of this recurrent CNV in the adult population of ID and comorbid psychiatric disorder. The yield of VOUS CNVs in the study also appears to be high at 62%. This is of considerable interest for future research, although it is difficult to determine whether VOUS are enriched in this cohort due to lack of comparable data sets.

A broad range of psychiatric diagnoses/symptoms were observed across the cohort. The pattern of comorbidities, either defined by ICD-10 diagnoses or Mini PAS-ADD thresholds, was complex. Inclusion of Mini PAS-ADD thresholds indicated a burden of psychopathology not captured by ICD-10 diagnoses. It is of interest that 41% of participants with likely pathogenic CNVs met the Mini PAS-ADD threshold for psychosis, whereas based upon ICD-10 criteria this was only 14%, see Table 2. Overall psychiatric features did not differentiate the likely pathogenic and likely benign CNV groups. However there was an excess of participants on a forensic section in the likely pathogenic CNV group in comparison with the benign (including VOUS) CNV group. No link can be made between specific CNVs and offending behaviour, as causality cannot be inferred. The population of people with ID and convictions is particularly marginalised and it may be that increased barriers to genetic testing have resulted in a higher prevalence of undiagnosed CNVs. This finding warrants further investigation in much larger samples. Assessment of adults with ID and formulation of the psychiatric presentation can be challenging. The majority of likely pathogenic CNVs were found at recurrent CNV loci where, at least some, information on the associated phenotype is available. Such information may aid understanding of the patient’s clinical presentation for both clinicians and family members. Furthermore knowledge of associated phenotypes may guide psychiatric evaluation. For example the identification of a 16p11.2 duplication would be an indicator to screen for the presence of ASD, psychosis or affective disorders.^15^ Importantly some of the CNV syndromes are also associated with non-psychiatric comorbidities that require specific medical management or monitoring. For example we identified an individual with undiagnosed Angelman’s syndrome type 2 and an individual with an *NF1* deletion. Comprehensive medical guidelines are available for Angelman’s syndrome and *NF1* deletions.^30^ The characterisation of very rare CNVs is an ongoing process and clinical guidelines are being developed as new syndromes continue to emerge. However, some diagnoses will have minimal impact on patient management.

There is evidence for the benefit to mothers in receiving a diagnosis for a child with DD/ID.^31^ However, little is known about the impact of disclosure of a genetic diagnosis to adults with ID or their families/carers. Challenges are faced with ascertaining capacity to consent to genetic testing, feedback of diagnoses and possible incidental findings. We received no negative feedback from psychiatrists in this study about the diagnostic process. All participants with likely pathogenic CNVs were sent information about a rare chromosomal support group called Unique; this group is only accessible to individuals with a diagnosis.^14^ Genetic diagnosis of the affected individual provides the opportunity for cascade testing of at risk relatives and the provision of recurrence risk information. The inheritance of a likely pathogenic CNV in a child with associated difficulties may be important in supporting an application for a statement of special educational needs.

This study has several specific limitations. The sample size was modest. The recruitment strategy focused on individuals with a more severe psychiatric phenotype, that is, those presenting to psychiatric services. We may have under sampled those with the most severe phenotypes because of difficulties recruiting this population group to research studies. However, our sample is likely representative of individuals accessing specialist services for ID and comorbid mental illness. Estimates of the penetrance of particular phenotypes would require epidemiological based studies. Another major limitation is the lack of inheritance status, which is often used to inform interpretation of rare variants. Technological limitations of the aCGH platform include inability to detect balanced translocations, single-gene disorders and low-level mosaicism. As the array platform has not been utilised in research studies of control populations comparisons with other studies is prone to technical confounds. Exome and whole-genome sequencing, which is gradually coming into clinical practice, is likely to increase the yield of genetic diagnoses for those with ID and comorbid psychiatric disorders.

## Conclusions

We have demonstrated that CNV screening using clinically available CMA offers over one in ten new aetiological diagnoses for adults with idiopathic ID presenting to psychiatric services in the UK. It may be appropriate for ID psychiatrists to offer CMA more routinely in assessment of people with ID and comorbid mental health problems, particularly in forensic settings. Liaison between clinicians and genetic services will be important for the interpretation of new genomic investigations in clinical practice.^32^

Clinical and research data on emerging CNV syndromes are strongly biased towards paediatric populations. However, the full extent of the phenotype associated with a particular CNV may only be realised in adulthood as psychiatric disorders emerge. We found the 16p11.2 duplication to be particularly frequent in this understudied adult population. We also add psychiatric phenotypic information to very rarely observed and novel likely pathogenic CNVs. CMA testing in adults could potentially inform the clinical management of children with new emerging likely pathogenic CNVs.

## Figures and Tables

**Table 1:** Likely pathogenic CNVs and psychiatric phenotypes in adults with ID referred to psychiatric services

*Decipher ID*	*Cytogenetic location*	*Gain/loss*	*Chromosomal region*	*Gender*	*Age*	*Level of ID*	*Psychiatric history*	*Mini PAS-ADD*	*BPI-S*			*Ethnicity*
327138	2p16.3	Loss	hg19 chr2:g.(51127940_51196188)_(51745532_51905988)del	Female	21	Moderate	PD	PSY, UNS	—	—	+	White (British)
327136	2q13	Loss	hg19 chr2:g.(111343032_111394464)_(113101432_113115816)del	Male	19	Mild	ASD	ANX, PSY	+	+	+	White (British)
327134	4p16.3	Gain	hg19 chr4:g.(?_116643)_(2593265_2641554)dup	Male	33	Mild	ASD, ALC, FOR	NA	NA			White (British
327131	6p25.3-24.3^a^	Gain	hg19 chr6:g.(?_195438)_(7391605_7414866)dup	Male	49	Mild	ANX, DEP	HYP, PSY, ASD	—	+	+	White (British)
327120	12p13.1	Gain	hg19 chr12:g.(13749971_13754557)_(13762818_13809913)dup	Female	68	Moderate	CB	None met	+	+	—	White (British)
327126	12q21.2-21.31	Loss	hg19 chr12:g.(79480496_79534648)_(84535824_84597248)del	Male	31	Mild	SCZ, ALC, FOR	HYP, PSY	—	—	—	Black (African)
327125	13q32.3-33.3	Gain	hg19 chr13:g.(100448328_100465760)_(109578072_109617112)dup	Male	21	Mild	ASD, ADHD, FOR	None met	—	—	—	White (British)
327128	15q11.2	Loss	hg19 chr15:g.(22728696_22759716)_(23071814_23149168)del	Female	42	Moderate	BP	DEP, ANX, HYP, OCD, PSY, UNS	—	—	+	White (British)
327127	15q11.2-13.1	Loss	hg19 chr15:g.(23510712_23643100)_(28519142_28565662)del	Female	33	Severe	ANX	ANX	+	—	+	White (British)
327124	15q11.2-13.1	Gain	hg19 chr15:g.(23780822_23810484)_(28519142_28565662)dup	Male	22	Mild	ASD, BP, PD, ALC, FOR	NA	—	+	—	White (British)
327123	15q12-13.1	Loss	hg19 chr15:g.(26445256_26455764)_(29576872_29582436)del	Male	28	Severe	ASD, ADHD	OCD, ASD	+	+	+	White (Other)
327137	15q13.2-13.3	Loss	hg19 chr15:g.(30357676_30461190)_(32804210_32906806)del	Male	25	Mild	ASD^b^	None met	—	—	—	White (British)
327122	16p11.2	Gain	hg19 chr16:g.(29387604_29443984)_(30192562_30351380)dup	Female	33	Mild	AFF	DEP, ANX, PSY	—	—	—	White (British)
327119	16p11.2	Gain	hg19 chr16:g.(29565700_29746324)_(30093468_30192562)dup	Female	27	Mild	ASD, DEP, OCD	DEP, OCD	—	—	—	White (Other)
327121	16p11.2	Gain	hg19 chr16:g.(29565700_29746324)_(30093468_30192562)dup	Female	62	Mild	DEP (psychotic)	PSY	—	—	—	White (British)
327133	16p11.2	Gain	hg19 chr16:g.(29565700_29746324)_(30192562_30351380)dup	Male	21	Mild	ASD, ADHD, FOR	None met	—	+	—	White (British)
327135	16p11.2	Loss	hg19 chr16:g.(29565700_29746324)_(30192562_30351380)del	Male	19	Mild	None recorded	DEP, ANX, HYP, OCD, PSY	—	—	+	White (British)
327130	16p13.11	Gain	hg19 chr16:g.(14880575_14892215)_(16616426_16625074)dup	Female	45	Moderate	CB	NA	NA			White (British)
327132	17q11.2	Loss	hg19 chr17:g.(29023540_29068144)_(30281856_30458900)del	Male	57	Severe	ASD, CB	ANX, HYP, OCD	+	+	+	White (British)
327131	18p11.32-11.31^a^	Loss	hg19 chr18:g.(122379_141491)_(6964201_7010334)del	Male	49	Mild	ANX, DEP	HYP, PSY, ASD	—	+	+	White (British)
327129	19q13.32	Loss	hg19 chr19:g.(45684624_45741740)_(47268136_47309140)del	Female	58	Mild	BP	DEP, PSY	—	+	—	White (British)
327139	Xq24-25	Gain	hg19 chrX:g.(118855888_118883248)_(123283296_123349096)dup	Male	57	Moderate	CB	None met	—	+	+	Black (Caribbean)
NA	XXYY	Gain	Full chromosomal duplications	Male	59	Mild	DEP, FOR	None met	—	+	—	White (British)

Chromosomal region coordinates in hg19 using the HGVS standard nomenclature; Age, age at date of recruitment; Level of ID, taken from medical records in accordance with the UK ICD-10 diagnostic system: 50–69, mild; 35–49, moderate; 20–34; severe. Mini PAS-ADD, Psychiatric Assessment Schedules for Adults with Developmental Disabilities thresholds met (in last 2 years); BPI-S, the Behaviour Problems Inventory-Short Form (self-injurious behaviour, aggressive/destructive, stereotyped) items scored as+when behaviour occurs at least weekly; PD, personality disorder; PSY, psychosis; UNS, unspecified disorder; ASD, autistic spectrum disorder; ANX, anxiety disorder; ALC alcohol abuse; FOR on forensic in-patient section; NA, not available; DEP, depression; HYP, hypomania/mania; CB, challenging behaviour; SCZ, schizophrenia; ADHD, attention-deficit hyperactivity disorder; BP, bipolar disorder; OCD, obsessive compulsive disorder; AFF, schizoaffective disorder.

a CNVs in the same participant.

b ASD traits only.

**Table 2:** Psychiatric phenotype (ICD-10 diagnoses, Mini PAS-ADD thresholds and section history) for likely pathogenic and benign CNVs

	*Total in sample (%)* n=*202*	*Likely pathogenic CNV group (%)* n=*22*	*Likely benign CNV group (%)* n=*180*
*ICD-10 diagnosis*			
Psychosis	49 (25%)	3 (14%)	46 (26%)
Bipolar disorder	23 (11%)	3 (14%)	20 (11%)
Depressive episode	62 (31%)	4 (18%)	58 (32%)
Other anxiety disorders	45 (23%)	2 (9%)	43 (24%)
Hyperkinetic disorder	21 (10%)	3 (14%)	18 (10%)
Pervasive developmental disorder	68 (34%)	8 (36%)	60 (33%)
			
*Mini PAS-ADD thresholds*			
Psychosis	72 (36%)	9 (41%)	63 (35%)
Hypomania/mania	33 (16%)	5 (23%)	28 (16%)
Depressive disorder	76 (38%)	5 (23%)	71 (39%)
Anxiety disorder	80 (40%)	6 (27%)	74 (41%)
Obsessive compulsive	55 (27%)	5 (23%)	50 (28%)
			
*Mental Health Act Section History*			
Previous history of involuntary admission	45 (22%)	7 (32%)	38 (21%)
Forensic section	21 (10%)	6 (27%)	15 (8%)

ICD-10 diagnoses – the psychosis group was amalgamated to comprise: F20 schizophrenia, F25 schizoaffective disorder and F29 unspecified nonorganic psychosis. Other ICD-10 diagnoses reported independently are: F31 bipolar disorder, F32 depressive episode, F41 other anxiety disorders, F90 hyperkinetic disorder, F84 pervasive developmental disorder. Mini PAS-ADD thresholds – scores were calculated using standard guidelines, Mental Health Act (MHA) section – previous history of involuntary admission included previous and current MHA sections and forensic sections, forensic section – all individuals were on a forensic section at the time of recruitment no history of being on a forensic section was identified in any of the other participants. Note: several individuals had comorbid diagnoses and are included in more than one category.
