# Supplementary material for: Chromosomal microarray testing in adults with intellectual disability presenting with comorbid psychiatric disorders
Source: Eur J Hum Genet. 2016 Sep 21;25(1):66–72. doi: 10.1038/ejhg.2016.107 (PMC5159755; doi:10.1038/ejhg.2016.107)
Supplement: Supplementary Information [file ejhg2016107x1.doc]

**Table 3: Medical phenotype and dysmorphic features in participants with emerging rare neurodevelopmental CNVs**

| **Decipher**  **ID** | **Cytogenetic Location** | **Age** | **Medical history** | **Head circumference (Cm)** | **Height (Cm)** | **Dysmorphic features** |
| --- | --- | --- | --- | --- | --- | --- |
| 327138 | 2p16.3 | 21 | Asthma | 55.9 | 169 | Abnormal facial shape, dental crowding |
| 327136 | 2q13 | 19 | Recurrent ear infections, urinary reflux, facial nerve palsy | N/A | N/A | Cranial abnormality |
| 327134 | 4p16.3 | 33 | Constipation | 59 | 179 | Upward slanting palpebral fissures, macrotia, prognathia |
| 327131 | 6p25.3-24.3 +  18p11.32-11.31 | 49 | Bilateral sensorineural hearing impairment, epilepsy, psychogenic polydipsia, hypogonadism, arthritis, osteoperosis, dysphagia | 56 | 157 | Facial asymmetry, abnormal facial shape, dental crowding, abnormality of the fingers, large ears, cranial abnormality |
| 327120 | 12p13.1 | 68 | Pes cavus (requiring callipers), gastric reflux | 57 | 152 | No gross dysmorphology |
| 327126 | 12q21.2-21.31 | 31 | None recorded | 64 | 169 | Hypertelorism, depressed nasal bridge, wide nasal bridge, low set ears, microtia |
| 327125 | 13q32.3-33.3 | 21 | Epilepsy, shuffling gait, bradykinesia | 59 | 167 | Low set ears, abnormality of the hand |
| 327128 | 15q11.2 | 42 | Recurrent urinary tract infections | 55 | N/A | Abnormality of external nose, abnormalities of the fingers |
| 327127 | 15q11.2-13.1 | 33 | Hypotonia (infant), epilepsy (infancy), probable diplopia, abnormality on neuroimaging | 53 | 161 | Upward slanting palpebral fissures, prognathia, protruding tongue, hypopigmentation of the skin |
| 327124 | 15q11.2-13.1 | 22 | None recorded | N/A | 182 | No gross dysmorphology |
| 327123 | 15q12-13.1 | 28 | Epilepsy (grand mal and absence seizures) | 55.8 | 170 | Cranial abnormality, facial asymmetry |
| 327137 | 15q13.2-13.3 | 25 | Type II diabetes | 60 | 173 | No gross dysmorphology |
| 327122 | 16p11.2 | 33 | Myopia | 57.9 | 173.4 | No gross dysmorphology |
| 327119 | 16p11.2 | 27 | Renal problems (childhood), menorrhagia, anaemia (severe), onychogryphosis | 54.2 | 161.8 | No gross dysmorphology |
| 327121 | 16p11.2 | 62 | Jaundice (childhood), epilepsy (childhood), type II diabetes, constipation, glaucoma | 53.2 | 172.5 | No gross dysmorphology |
| 327133 | 16p11.2 | 21 | Insulin dependent diabetes, hypercholesterolaemia | 59 | 188 | N/A |
| 327135 | 16p11.2 | 19 | Seizures (infancy), acne | 59 | 162 | Tapering fingers |
| 327130 | 16p13.11 | 45 | Hypertension, type II diabetes, constipation, asthma, obesity | 54 | 147 | Abnormal facial shape, microtia |
| 327132 | 17q11.2 | 57 | Pacemaker in situ (long Q-T syndrome), hypothyroidism, hypercholesterolaemia, cataracts | 60 | 179 | Abnormalities of the fingers |
| 327131 | 18p11.32-11.31 | 49 | Bilateral sensorineural hearing impairment, epilepsy, psychogenic polydipsia, hypogonadism, arthritis, osteoperosis, dysphagia | 56 | 157 | Facial asymmetry, abnormal facial shape, dental crowding, abnormality of the fingers, large ears, cranial abnormality |
| 327129 | 19q13.32 | 58 | Epilepsy, incontinence, Cataracts, broad based gait | 55 | 148 | Short upturned nose |
| 327139 | Xq24-25 | 57 | Shuffling gait, bradykinesia | 61 | 174 | Abnormality of the skull, abnormality of the eyelid, abnormal nasal morphology, abnormalities of the fingers |
| Not applicable | XXYY | 59 | Hypertension, hypercholesterolaemia, type I diabetes, absent kidney, constipation, cataracts, anaemia, asthma, osteoarthritis | 58.5cm | 176cm | N/A |

Age, age at date of recruitment ; Medical history was collected using a standard pro-forma from informants (family members and/or carers) and medical records where available. Dysmorphic features were recorded using a standard pro-forma by researchers carrying out the assessment. Only dysmorphic features obvious on general observation in normal clothing were documented no structured physical examination was undertaken. Dysmorphic features were corroborated by examination of accompanying photographs by the study team. Not all participants consented to be photographed; N/A, Not Available.
